# Inhibition of IRE1α RNase activity reduces NLRP3 inflammasome assembly and processing of pro-IL1β

**DOI:** 10.1038/s41419-019-1847-z

**Published:** 2019-08-16

**Authors:** Aaron Talty, Shane Deegan, Mila Ljujic, Katarzyna Mnich, Serika D. Naicker, Dagmar Quandt, Qingping Zeng, John B. Patterson, Adrienne M. Gorman, Matthew D. Griffin, Afshin Samali, Susan E. Logue

**Affiliations:** 1 Apoptosis Research Centre, National University of Ireland Galway, Galway, Ireland; 2 School of Natural Sciences, National University of Ireland Galway, Galway, Ireland; 3 Regenerative Medicine Institute (REMEDI) at CÚRAM Centre for Research in Medical Devices, School of Medicine, College of Medicine, Nursing and Health Sciences, National University of Ireland Galway, Galway, Ireland; 4 Institute of Anatomy and Cell Biology, Faculty of Medicine, Martin Luther University Halle-Wittenberg, Halle (Saale), Germany; 5 Fosun Orinove PharmaTech Inc., Suite 211, Building A4, 218 Xinghu St., Suzhou Industrial Park, 215123 Jiangsu, China; 6 Fosun Orinove PharmaTech Inc., 3537 Old Conejo Road, Suite 104, Newbury Park, CA 91320 USA; 7 Department of Human Anatomy and Cell Science, Rady Faculty of Health Sciences, Max Rady College of Medicine, University of Manitoba, Winnipeg, MB Canada

**Keywords:** Stress signalling, Inflammasome

## Abstract

The inflammasome is a multiprotein complex assembled in response to Pathogen Associated Molecular Patterns (PAMPs) and Danger Associated Molecular Patterns (DAMPs). Inflammasome activation occurs through a two-step mechanism, with the first signal facilitating priming of inflammasome components while the second signal triggers complex assembly. Once assembled, the inflammasome recruits and activates pro-caspase-1, which in turn processes pro-interleukin (IL)-18 and pro-IL-1β into their bio-active forms. Owing to its key role in the regulation of innate immune responses, the inflammasome has emerged as a therapeutic target for the treatment of inflammatory conditions. In this study we demonstrate that IRE1α, a key component of the Unfolded Protein Response, contributes to assembly of the NLRP3 inflammasome. Blockade of IRE1α RNase signaling lowered NLRP3 inflammasome assembly, caspase-1 activation and pro-IL-1β processing. These results underscore both the importance and potential therapeutic relevance of targeting IRE1α signaling in conditions of excessive inflammasome formation.

## Introduction

Protein folding and processing of transmembrane and secretory proteins occur within the endoplasmic reticulum (ER). Exposure to physiological or pathological conditions, such as infections, nutrient deprivation, or hypoxia, reduces the ability of the ER to function leading to a build-up of unfolded or misfolded proteins—a condition referred to as ER stress. The accumulation of unfolded/misfolded proteins activates three ER-anchored transmembrane receptors—inositol-requiring enzyme 1α (IRE1α)^1,2^, protein kinase RNA (PKR)-like ER kinase (PERK)^3^, and activating transcription factor-6 (ATF6)^4^—triggering a signaling cascade termed the unfolded protein response (UPR). The UPR is an evolutionarily conserved stress response pathway tasked with reducing levels of unfolded/misfolded proteins and returning homeostasis to the ER^5^.

IRE1α, a bifunctional transmembrane protein comprised of both a serine/threonine kinase domain and an endoribonuclease (RNase) domain, is the most evolutionarily conserved UPR sensor present in all eukaryotes. Similar to PERK and ATF6, the N-terminus of IRE1α juts into the ER lumen where, under nonstress conditions, it associates with the ER chaperone glucose-regulated protein 78 (Grp78)^6^. Accumulation of unfolded proteins initiates Grp78 dissociation from IRE1α permitting IRE1α dimerization and trans-autophosphorylation leading to activation of its RNase domain. IRE1α RNase activity has two principal outputs; splicing of a 26-nucleotide intron from *X-box binding protein 1* (*XBP1)* mRNA^7^ and IRE1α-dependent decay of mRNA (RIDD), the sequence specific cleavage of non-*XBP1* mRNAs and selected miRNAs localized to the ER membrane^8,9^. Splicing of *XBP1* mRNA leads to the translation of a stable transcription factor termed XBP1s, which regulates expression of ER chaperones and components of the ER-associated degradation machinery thus enhancing the capacity of the ER to reduce unfolded/misfolded proteins^10,11^.

Although IRE1α has a well-established role in the UPR, its influence may extend beyond monitoring ER homeostasis. IRE1α signaling has been demonstrated to contribute to the development of several immune cell types, including secretory plasma cells^12^ and dendritic cells^13^. IRE1α-mediated regulation of macrophage polarization under conditions of metabolic stress has also been reported^14^. In addition to immune cell development, several studies have also demonstrated that IRE1α-XBP1 signaling contributes to innate immune responses triggered by various toll-like receptor (TLR) ligands including lipopolysaccharide (LPS)^15^, attenuated *Brucella abortus* strain^16^ and Methicillin-resistant *Staphylococcus aureus* (MRSA) infection^17^. Furthermore, IRE1α activity was also shown to be upregulated in inflammatory arthritis^18^ as well as in lipid-induced inflammation^19^.

In this study, we examined the contribution of IRE1α RNase activity to inflammasome formation and in particular the nucleotide-binding oligomerization domain, leucine rich repeat and pyrin domain containing 3 (NLRP3) inflammasome. Structurally, the NLRP3 inflammasome is composed of three components—NLRP3 that functions as a sensor protein; the adapter apoptosis-associated speck-like protein containing a caspase recruitment domain (ASC) and pro-caspase-1^20^. Activation is achieved via a two-step mechanism with the first step (priming step) involving transcriptional upregulation of key components including NLRP3 as well as pro-IL-1β through TLR activation and subsequent NF-κB signaling^21,22^. The second step (signal II) promotes NLRP3 inflammasome assembly and activation. The precise mechanisms facilitating NLRP3 inflammasome activation remain unclear with several models proposed^23–25^. Ultimately, signal II enables structural assembly of the inflammasome with NLRP3 recruiting ASC via pyrin:pyrin domain interactions, which in turn triggers ASC oligomerisation leading to the formation of long ASC filaments^26^. Pro-caspase-1 is recruited to ASC via CARD:CARD interactions leading to auto-processing resulting in the generation of activate caspase-1.

We now report that inhibition of IRE1α RNase activity, while not impacting on inflammasome priming, selectively reduces structural assembly of the inflammasome. This suggests that small molecule inhibitors of IRE1α RNase activity may offer a new therapeutic opportunity for diseases mediated by excessive or prolonged NLRP3 inflammasome activity.

## Material and methods

### Antibodies and reagents

Primary antibodies used were as follows: mouse anti-XBP1s (Biolegend, 647502), rabbit anti-IRE1 (Cell Signaling, 3294), rabbit anti-PERK (Cell Signaling Technology, 3192), rabbit anti-eIF2α (Cell Signaling Technology, 5324), rabbit anti-p(S51)-eIF2α (Cell Signaling Technology, 3398), mouse anti-ATF6 (CosmoBio, BAM-73-500-EX), rabbit anti-NLRP3 D2P5E (Cell signaling technology, 13158), rabbit anti-NF-κB p65 (D14E12) (Cell signaling technology, 8242), mouse anti-IL-1β (R&D Systems, MAB601), rabbit anti-ASC (Santa Cruz, sc-22514-R), rabbit anti-caspase 1 (Santa Cruz, sc-622), rabbit anti-caspase-1 p10 (Santa Cruz, sc-515), TXNIP (Santa Cruz, sc-166234), and rabbit anti-Actin (Sigma, A2066). Secondary antibodies were horseradish peroxidase-tagged goat anti-mouse (Jackson Laboratories, 115-035-003) and goat anti-rabbit antibodies (Jackson Laboratories, 111-035-003). Tunicamycin (T7765), Phorbol 12-myristate 13-acetate (PMA) (P8139), LPS (L2630), and ATP (A6419) were purchased from Sigma-Aldrich while nigericin (tlrl-nig) was obtained from Invivogen. IRE1 inhibitor MKC8866 was provided by Fosun Orinove.

### Cell culture

THP-1 cells were purchased from ATCC and cultured in RPMI 1640 media (Sigma, R0883) supplemented with 10% heat-inactivated fetal bovine serum (Sigma, F7524) and 2 mM l-glutamine (Sigma, G7513). Blood sampling of healthy volunteers was carried out following informed consent at the National University of Ireland, Galway (NUI Galway) under a protocol entitled “Immunological research using healthy human blood cells” approved by the NUIG Research Ethics Committee on 30/4/14 (Protocol no. 14/MAR/01). Human peripheral blood mononuclear cells (PBMCs) were isolated by Ficoll-Hypaque density gradient centrifugation from freshly drawn EDTA-anticoagulated peripheral venous blood. Briefly, 3 ml aliquots of EDTA-anticoagulated peripheral venous blood was layered over 3 ml of Ficoll Paque Plus (Sigma, GE17-1440-02) in a 15 ml tube and centrifuged at 400 × *g* for 22 min at 4 °C. The thin cloudy layer of PBMCs present at the interface of plasma and red blood cell layers was removed and transferred to a fresh 15 ml tube. The PBMCs were washed in 10 ml FACS buffer (2% fetal calf serum, PBS and 0.05% NaN_3_) and were pelleted by centrifugation at 300 × *g* for 10 min at 4 °C. The supernatant was discarded and the cell pellet was resuspended in 5 ml FACS buffer and washed a second time using the same protocol. The final cell pellet was resuspended in 1 mL FACS buffer and counted using a haemocytometer. Freshly isolated PBMCs were diluted in complete media containing RPMI‐1640 (Sigma, R0883) supplemented with 1% l‐glutamine (Sigma, G7513) 100 U/ml penicillin/100 mg/ml streptomycin (Sigma, P0781) and 5% clotted male human AB serum (Sigma, H6914). All cells were grown at 37 °C and 5% CO_2_.

### Transfections

Prior to transfection THP-1 cells were treated with PMA (50 ng/ml) for 24 h following which they were transfected with siRNA using TransIT-TKO (Mirus, MIR 2154) and according to the manufacturer’s protocol. siRNAs (ON-TARGET plus smart pool) ERN1/IRE1 (L-004951-02-0005); non-coding siRNA (D-001810-10-20) was obtained from Dharmacon. The media was changed 6 h post transfection and cells were left to recover for 72 h.

### Inflammasome activation

THP-1 cells were plated in six-well plates at a density of 1 × 10^6^ cells/ml and treated with 1 μg/ml LPS with or without IRE1 inhibitor MKC8866 (20 μM) for 24 h. After 24 h, LPS primed cells were treated with 10 μM nigericin for 45 min following which cells and conditioned medium were collected for analysis. To suppress NLRP3 inflammasome activation, 100 mM KCl was added to the culture medium at the time of LPS addition. For inflammasome activation, PBMCs were plated at a density of 1 × 10^6^ cells/ml and primed with 0.5 ng/ml LPS for 2 h in the presence or absence of MKC8866 (20 μM). After 2 h of priming 5 mM ATP was added for 45 min and cells and conditioned medium were collected for analysis.

### ASC crosslinking

Following treatment THP-1 cells were resuspended in 500 μl of ice-cold buffer (20 mM HEPES-KOH, pH 7.5, 150 mM KCl, 1 mM Na_3_VO_4_, 0.1 mM PMSF, 1% NP-40, and a protease inhibitor cocktail) and lysed by shearing ten times using a 21 gauge needle. Lysates were centrifuged at 300 × *g* for 5 min at 4 °C. Supernatants were filtered through a 5 μM filter (GE Healthcare, 6784-1350). Filtrates were centrifuged at 6800 × *g* for 15 min at 4 °C to pellet ASC-insoluble specks. Supernatants were transferred to new tubes (ASC-soluble fractions). The ASC-insoluble pellets were washed with PBS twice and then suspended in 200 μl PBS. The ASC-insoluble pellets were cross-linked at room temperature for 30 min by adding 2 mM bis[sulfosuccinimidy]suberate (BS3). The solutions were then centrifuged at 6800 × *g* for 15 min at 4 °C to pellet the cross-linked ASC which was then dissolved directly in SDS sample buffer.

### Caspase-1 activity assay

THP-1 cells were treated as indicated and whole-cell caspase-1 activity was determined using the FLICA 660 in vitro Caspase-1 detection Kit (Immunochemistry Technologies, 9122) by the use of flow cytometry. Cell suspensions were aliquoted into 96‐well flat‐bottom cell culture plates at a final concentration of 1 × 10^6^ cells/ml in complete medium and a total incubation volume of 200 μl. At the time of signal II addition, 10 μl of FLICA reagent (reconstituted as directed by manufacturer and diluted to 1:10 in FACS buffer 5 min prior to staining) was added to each well. The cells were incubated for 45 min at 37 °C, 5% CO_2_ then washed using 1 ml PBS and pelleted by centrifugation at 300 × *g* for 5 min at 4 °C. Live cells were analysed on a BD Accuri C6 Flow Cytometer (BD Biosciences).

### Cytokine analysis

Conditioned medium was collected from treated THP-1 cells and PBMCs and assayed for cytokines using sandwich ELISA according to the manufacturer’s instructions (DuoSet, R&D Systems). Cytokines analysed included IL-1β (DY201-05), TNF-α (DY210-05), IL-6 (DY206-05), and CXCL8/IL-8 (DY208-05).

### RNA extraction, PCR and Q-PCR

RNA extraction from THP-1 cells and PBMCs was performed using the RNeasy micro kit (Qiagen) according to the manufacturer’s protocol, and cDNA was generated from 250 ng of RNA using Superscript III first strand RT-PCR system and random hexamers (Invitrogen). Semiquantitative RT-PCR was performed using GoTaq green master mix (Promega, M7123) with primers specific for *XBP1s* (Forward: 5′-TCTGCTGAGTCCGCAGCAGG-3′ and reverse: 5′-CTCTAAGACTAGAGGCTTGG-3′) and the endogenous control *GAPDH* (Forward: 5′-ACCACAGTCCATGCCATC-3′ and reverse: 5′-TCCACCACCTGTTGCTG-3′). Q-PCR reactions were performed using Takyon ROX Master Mix (Eurogentec UFRP5XC0501) and the StepOne Plus platform (Applied Biosystems). Target transcript levels were normalized to *GAPDH*, and relative abundance was determined using the ΔΔCt method. Transcript-specific TaqMan assays were purchased from Integrated DNA Technologies. The sequences of primers and probes used are detailed in the table below.

**Table:** 

*NLRP3*
* Probe*: 5′-TGCAGGTTACACTGTGGATTCTTGGC-3′
* Primer 1*: 5′-AGATTCTGATTAGTGCTGAGTACC-3′
* Primer 2*: 5′-GAATGCCTTGGGAGACTCAG-3′
*IL-1β*
* Probe:* 5′-AGAAGTACCTGAGCTCGCCAGTGA-3′
* Primer 1*: 5′-GAACAAGTCATCCTCATTGCC-3′
* Primer 2*: 5′-CAGCCAATCTTCATTGCTCAAG-3′
*Caspase-1*
* Probe:* 5′-AGTCTTCCAATAAAAACAGAGCCCATTGTG-3′
* Primer 1:* 5′-CACATCACAGGAACAGGCATA-3′
* Primer 2*: 5′-TGAAGGACAAACCGAAGGTG-3′
*TXNIP*
* Probe*: 5′ TTGCGGAGTGGCTAAAGTGCTTTG-3′
* Primer 1*: 5′-GTGATAGTGGAGGTGTGTGAAG-3′
* Primer 2*: 5′-CAGGTACTCCGAAGTCTGTTTG-3′
*GAPDH*
* Probe: 5*′*-AAGGTCGGAGTCAACGGATTTGGTC-3*′
* Primer 1: 5*′*-ACATCGCTCAGACACCATG-3*′
* Primer 2:* 5′-*TGTAGTTGAGGTCAATGAAGGG-3*′

### Immunoblotting

Protein samples lysed in 1× SDS-PAGE (2% sodium dodecyl sulfate (SDS), 50 mM Tris-HCl pH 6.8, 5% glycerol, 0.05% bromphenol blue, 357 mM β-mercaptoethanol) or RIPA (0.1% SDS, 1% NP-40, 0.5% sodium deoxycholate, 50 mM Tris-HCl pH 8.8, 150 mM NaCl) buffer were separated by SDS-PAGE and blotted onto nitrocellulose membranes (Amersham). Membranes were blocked with 5% nonfat dry milk in PBS-0.1% Tween 20 and then incubated at 4 °C overnight with primary antibodies specified above. Next day membranes were incubated with corresponding secondary horseradish peroxidase or IRDye–conjugated antibodies for 1 h at room temperature and bands were visualized using ECL Western blotting detection reagents (Amersham, GE Healthcare) or scanned using the Odyssey Infrared Imaging System (Li-Core Biosciences).

### Propidium iodide assessment of cell death

THP-1 cells were plated in a 12-well plate at a density of 1 × 10^6^ cells/ml and treated with 1 μg/ml LPS with or without indicated concentrations of MKC8866 for 24 h. After 24 h, LPS primed cells were treated with 10 μM nigericin for 45 min prior to cell death analysis. Cells were pelleted at 4000 × *g* for 5 min and resuspended in 100 μl of ice-cold PBS. Five minutes prior to analysis, 1.5 μg/ml Propidium Iodide (PI) (Sigma, P4170) was added and PI uptake analysed using BD Accuri C6 flow cytometer (BD Biosciences).

### Statistical analysis

Data are expressed as mean ± SD for at least three independent experiments. Statistical analysis was performed using GraphPad Prism (GraphPad Software, San Diego, CA). Significance was determined using a Student’s *t* test, with *P* < 0.05 being considered significant and annotated by *P < 0.05, **P < 0.001, ***P < 0.0001, and ****P < 0.00001.

## Results

### IRE1α RNase activity is selectively activated upon TLR4 stimulation

**Fig. 1:**
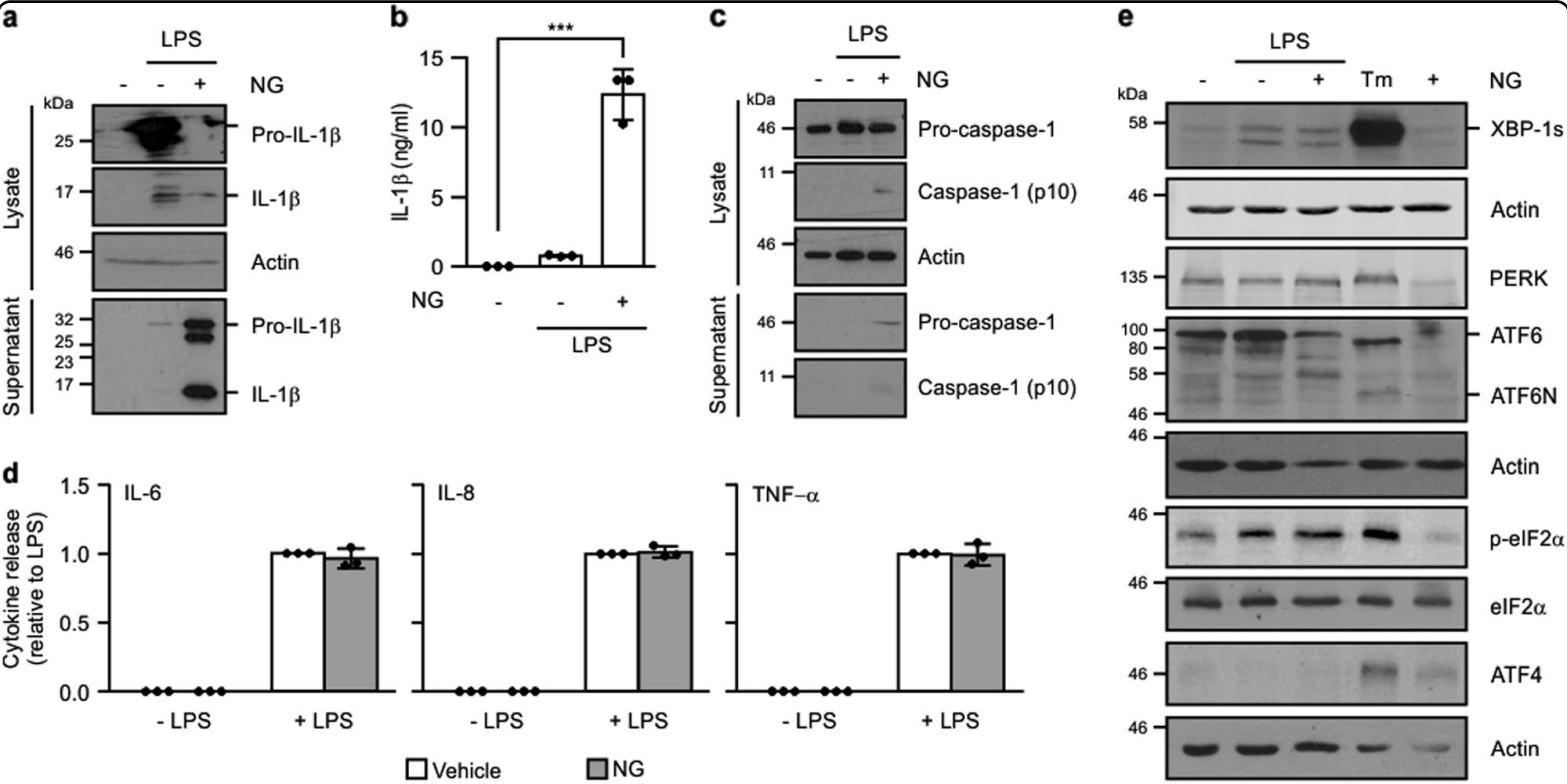
IRE1α-XBP1 axis of the UPR is selectively activated upon TLR4 stimulation. THP-1 cells were primed with either 1 μg/ml LPS alone for 24 h or 1 μg/ml LPS for 24 h followed by addition of 10 μM nigericin (NG) for 45 min. **a** Processing of pro-IL-1β was analysed in cell lysates and conditioned medium by immunoblotting for full-length pro-IL-1β and processed p17 IL-1β. **b** Levels of IL-1β were quantified in conditioned medium from untreated, LPS, LPS and NG-treated THP-1 cells by ELISA (*n* = 3). **c** Processing of caspase-1 was analysed in cell lysates and conditioned medium by immunoblotting for pro-caspase-1 and processed p10 caspase-1. **d** IL-6, IL-8, and TNF-α levels were quantified in conditioned medium from untreated, LPS, LPS and NG, and NG-only treated THP-1 cells by ELISA (*n* = 3). **e** UPR markers XBP1s, PERK, ATF6, phospho-eIF2α, total eIF2α, and ATF4 were analysed by immunoblotting in THP-1 post treatment with LPS alone or LPS and NG. Tunicamycin (Tm)-treated THP-1 cells served as a positive control for the UPR activation. Actin was used as a loading control. ***P < 0.001 based on a Student’s *t* test. Error bars represent SD.

The human monocytic cell line, THP-1, was treated with LPS (signal I) alone or LPS followed by a brief incubation with the K^+^ ionophore, nigericin (signal II) to stimulate NLRP3 inflammasome assembly. Addition of nigericin enhanced processing and secretion of pro-IL-1β compared with cells treated with LPS alone (Fig. 1a, b). Likewise, a similar increase in pro-caspase-1 processing was observed following nigericin addition confirming inflammasome formation (Fig. 1c). Nigericin addition did not alter levels of IL-6, IL-8, or TNF-α in LPS-treated THP-1 cell conditioned medium (Fig. 1d).

To determine if UPR signaling was triggered during inflammasome formation we assessed activation of IRE1α, PERK, and ATF6 in THP-1 cells following LPS and LPS/nigericin treatment. In agreement with published data^15^, IRE1α activation, as determined by XBP1 splicing, was detected by immunoblotting in cell lysates following both LPS and LPS/nigericin treatment (Fig. 1e). Activation of ATF6 or PERK arms of the UPR was not detected following treatment with either LPS alone or with LPS/nigericin. However, processing of ATF6 as signified by the appearance of amino terminal fragment of ATF6 (ATF6N), and PERK activation (as indicated by an upshift in PERK molecular weight, indicative of phosphorylation, and induction of ATF4 expression) was readily detected following treatment with tunicamycin, a pharmacological inducer of ER stress (Fig. 1e). Collectively, these results indicate that (a) addition of nigericin post-LPS treatment stimulates inflammasome formation in THP-1 cells and (b) LPS treatment leads to selective activation of the IRE1α branch of the UPR.

### IRE1α signaling contributes to LPS/nigericin inflammasome activity

**Fig. 2:**
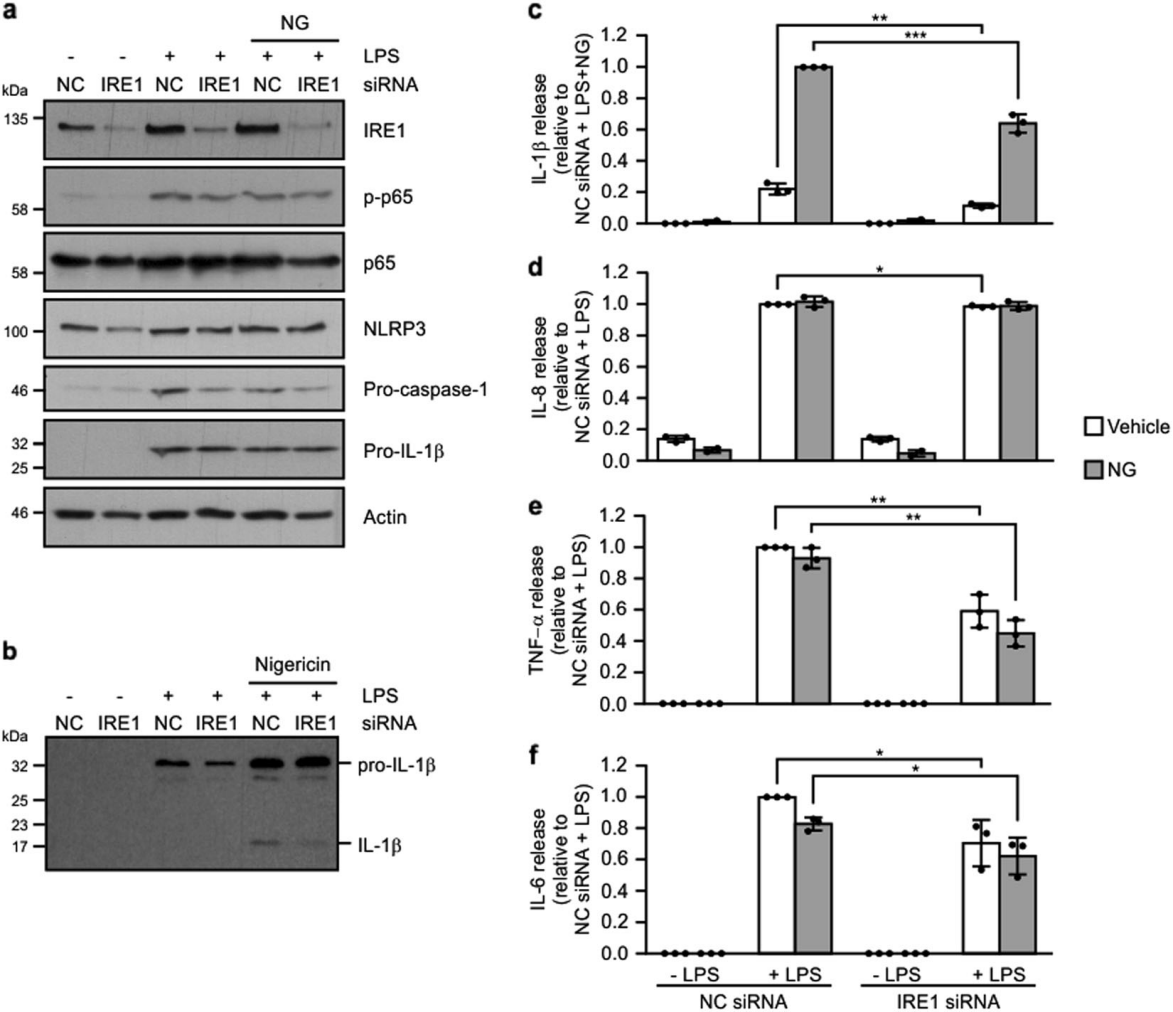
Knockdown of IRE1α reduces LPS-induced cytokine production. THP-1 cells transfected with either noncoding (NC) or IRE1α targeting siRNA were treated with either 1 μg/ml LPS alone for 24 h or 1 μg/ml LPS for 24 h followed by addition of 10 μM nigericin (NG) for 45 min. **a** Cell lysates were analysed via immunoblotting for IRE1α, phospho-p65, total p65, NLRP3, pro-caspase-1 and pro-IL-1β. Actin was used as a loading control. **b** Cell conditioned medium was analysed via immunoblotting for pro-IL-1β processing. **c**–**f** Levels of IL-1β, IL-8, TNF-α, and IL-6 were assayed in cell conditioned medium by ELISA (*n* = 3). *P < 0.05, **P < 0.01, and ***P < 0.001 based on a Student’s *t* test. Error bars represent SD.

To determine if IRE1α signaling actively contributes to NLRP3 inflammasome formation, or if it was merely a by-stander effect due to TLR4 activation, we transiently transfected THP-1 cells with siRNA targeting IRE1α. Efficient knockdown of IRE1α was verified by western blotting 72 h after siRNA transfection (Fig. 2a). Knockdown of IRE1α significantly reduced processing of pro-IL-1β and secretion of p17 IL-1β (Fig. 2b, c). In agreement with previous results^15^ assessment of IL-6 and TNF-α levels also indicated a role for IRE1α signaling with reduced IL-6 and TNF-α evident in conditioned medium from both LPS and LPS/nigericin-treated IRE1α siRNA transfected cells compared with their noncoding counterparts (Fig. 2d–f).

Recent reports have linked IRE1α, through its kinase activity, to initiation of NF-κB signaling^27^. Given the importance of NF-κB activation in mediating inflammasome activation we asked if knockdown of IRE1α dampened NF-κB signaling and, through this mechanism, reduced priming of essential components of the inflammasome such as NLRP3. While addition of LPS or LPS/nigericin clearly triggered NF-κB activation, as determined by an increase in phospho-p65 levels (Fig. 2a), this was not altered by IRE1α knockdown.

**Fig. 3:**
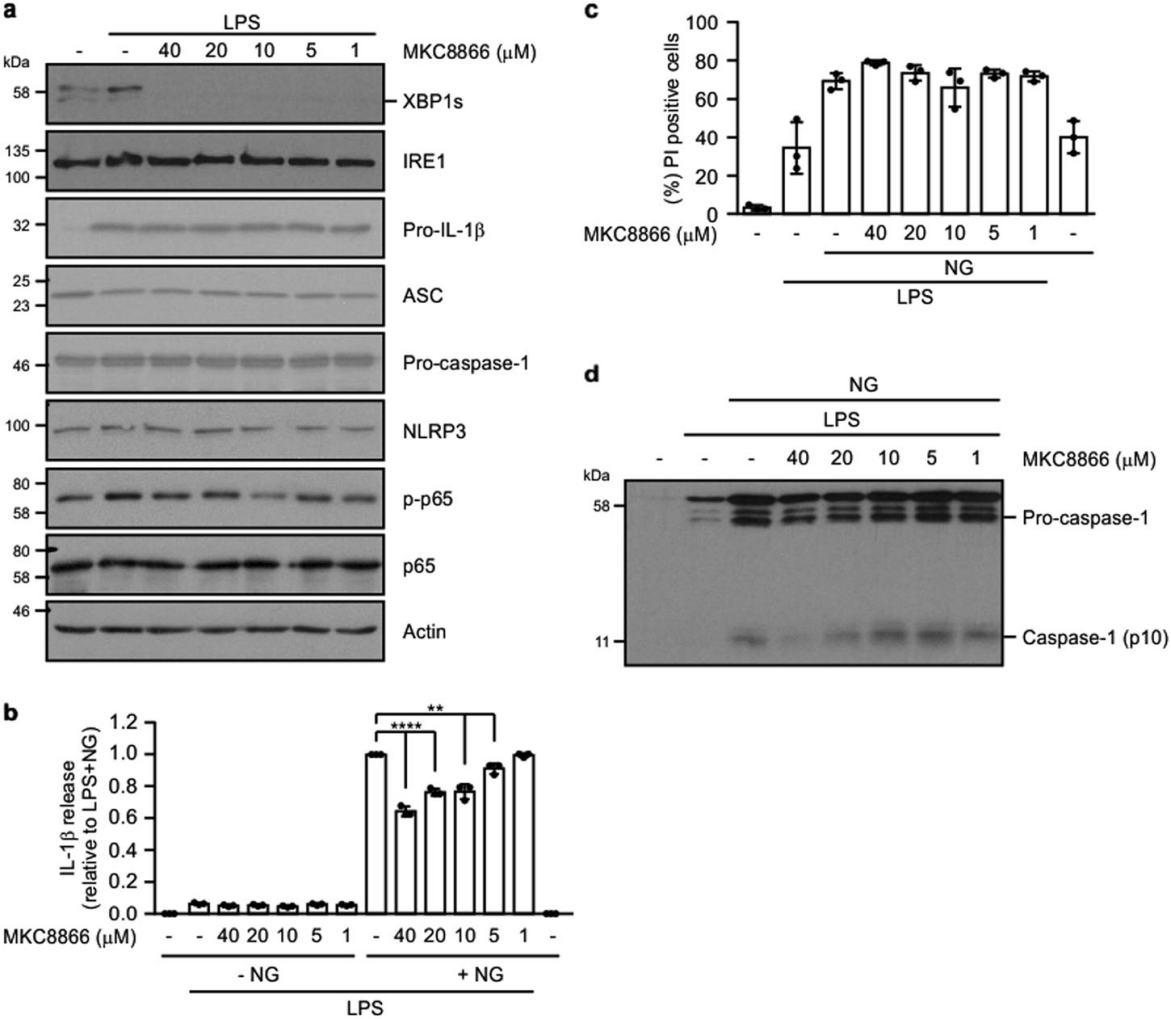
MKC8866 reduces LPS-induced IRE1α signaling and suppresses NLRP3 inflammasome activation. **a** THP-1 cells were treated with 1 μg/ml LPS alone or in combination with indicated concentrations of IRE1α inhibitor (MKC8866) for 24 h following which cell lysates were collected and analysed via immunoblotting for XBP1s, IRE1α, pro-IL-1β, ASC, pro-caspase-1, NLRP3, phospho-p65 and total p65. Actin was used as a loading control. **b** Conditioned medium from THP-1 cells treated with 1 μg/ml LPS alone or in combination with 10 μM nigericin (NG) in the presence or absence of indicated concentrations of MKC8866 were analysed by ELISA for IL-1β secretion (*n* = 3). **c** Propidium iodide (PI) uptake was assessed in THP-1 cells following treatment with 1 μg/ml LPS alone or in combination with 10 μM nigericin (NG) plus the indicated concentrations of MKC8866 (*n* = 3). **d** Conditioned medium from **b** was immunoblotted for pro- and cleaved caspase-1. **P < 0.01 and ****P < 0.0001 based on a Student’s *t* test. Error bars represent SD.

Owing to its structure and mode of activation IRE1α represents a druggable target. Indeed, over the past 6 years, a number of small molecule inhibitors selectively targeting IRE1α RNase activity have been reported. One such inhibitor, the salicylaldehyde analog MKC8866, is a selective, noncompetitive, potent, and reversible inhibitor of IRE1α RNase activity^28^. Treatment of THP-1 cells with MKC8866 efficiently abolished LPS-induced activation of IRE1α RNase signaling as demonstrated by a clear reduction in XBP1 splicing (Fig. 3a). Similar to IRE1α knockdown, MKC8866 significantly reduced levels of secreted IL-1β post-LPS/nigericin treatment in a dose-dependent manner (Fig. 3b).

Recent reports have suggested that release of IL-1β from cells following NLRP3 inflammasome activation is facilitated by signal II-induced cell death^29^. Based on these reports, we asked if the reduced IL-1β levels we observed in the conditioned medium of LPS/MKC8866/nigericin-treated THP-1 cells could be a consequence of decreased cell death. To answer this we assessed cell death, via propidium iodide (PI) uptake, in THP-1 cells treated with LPS alone or in combination with MKC8866. In agreement with recently published data^29^, we observed increased cell death following addition of signal II (nigericin) in LPS-stimulated cells. However, the level of cell death observed was similar in those cells treated with LPS alone and a combination of LPS and MKC8866 (Fig. 3c). This suggests that the reduced IL-1β levels we observed upon IRE1α inhibition is not a consequence of reduced cell death.

**Fig. 4:**
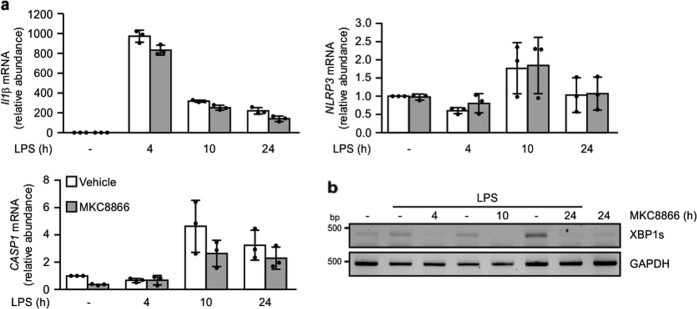
Addition of MKC8866 does not impact upon LPS-mediated increases in *CASP1*, *IL1β*, or *NLRP3* transcript. THP-1 cells were treated with 1 μg/ml LPS in the presence or absence of 20 μM MKC8866 for the indicated times after which RNA was extracted. **a** Q-PCR was carried out to assess relative expression of *IL1β* and *NLRP3* and *CASP1* transcripts (*n* = 3). Error bars represent SD. **b** RT-PCR was carried out to assess *XBP1* splicing and *GAPDH* mRNA expression.

Processing of pro-capsase-1 is a hallmark of NLRP3 inflammasome formation. Analysis of processed (p10) caspase-1 in conditioned medium from LPS/nigericin-treated THP-1 cells revealed a dose-dependent reduction associated with addition of MKC8866 (Fig. 3d). To understand how inhibition of IRE1α RNase activity reduced caspase-1 processing, we examined LPS-mediated regulation of inflammasome components in the presence and absence of MKC8866. Examination of pro-IL-1β, pro-caspase-1, ASC and NLRP3 protein expression in the lysate of LPS/nigericin-treated cells with and without MKC8866 did not show any alterations in their expression (Fig. 3a). In addition, we also examined transcript levels of each component over a 24 h LPS treatment. Following 4 h treatment with LPS, increases in pro-IL-1β transcript levels were apparent. By 10 h, elevated levels of NLRP3 and pro-caspase-1 transcripts were also observed verifying priming of the inflammasome (Fig. 4a). Similar to the results for protein expression, we observed no alteration in the transcript levels of pro-IL-1β, pro-caspase-1, ASC and NLRP3 upon MKC8866 addition (Fig. 4a). In contrast, there was a clear reduction in *XBP1s* expression, verifying MKC8866 functionality (Fig. 4b). This suggests that the suppression in pro-caspase-1 processing observed upon inhibition of IRE1α RNase activity is not a consequence of reduced expression of principal inflammasome components.

### Addition of MKC8866 reduces LPS/nigericin-induced inflammasome formation

**Fig. 5:**
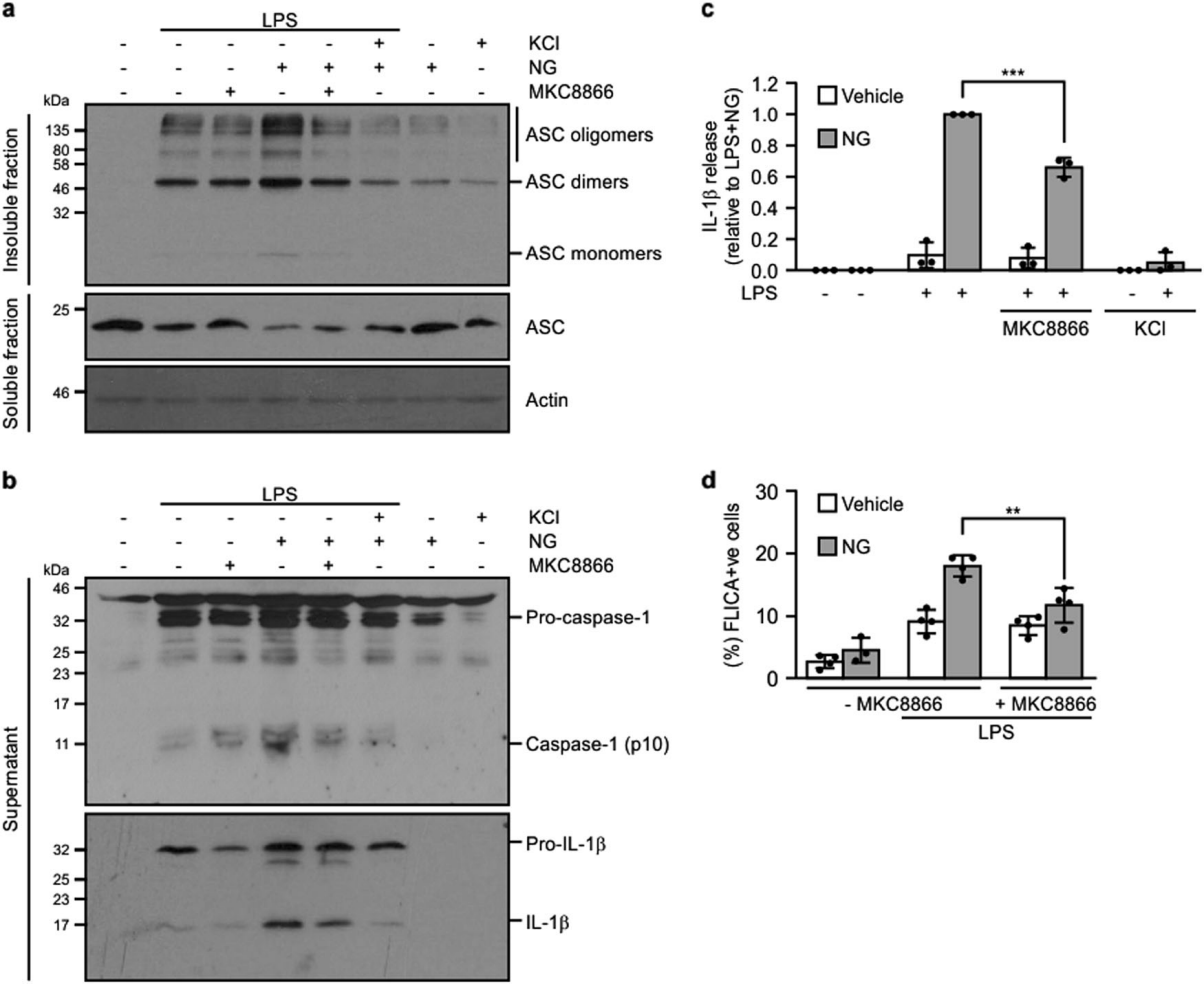
Addition of MKC8866 reduces NLRP3 inflammasome assembly. THP-1 cells were treated with either 1 μg/ml LPS alone or in combination with 20 μM MKC8866 for 24 h followed by addition of 10 μM nigericin (NG) alone or in combination with 100 mM KCl for 45 min after which cells and conditioned medium were harvested. Immunoblotting was used to determine **a** ASC oligomerization status and **b** caspase-1 (pro- and cleaved p10) and IL-1β processing. **c** IL-1β levels were quantified by ELISA in THP-1 conditioned medium following indicated treatments (*n* = 3). **d** The percentage of THP-1 cells displaying caspase-1-like activity following the indicated treatments was assessed by the FLICA assay (*n* = 4). **P < 0.01 and ***P < 0.001 based on a Student’s *t* test. Error bars represent SD.

To determine if the decreased pro-caspase-1 processing and pro-IL1β processing observed upon IRE1α inhibition was a consequence of reduced assembly of the inflammasome complex, we assessed ASC oligomerisation following LPS and LPS/nigericin treatment in the presence and absence of MKC8866. Treatment of THP-1 cells with LPS/nigericin triggered an increase in ASC oligomerization compared with LPS alone highlighting the need for signal II to potentiate inflammasome formation (Fig. 5a). As previously reported^23^, inclusion of high extracellular K^+^ negated the ability of nigericin to enhance ASC oligomerization **(**Fig. 5a**)**. While combination with MKC8866 substantially reduced ASC oligomerization, it was not to the extent observed with high extracellular K^+^ (Fig. 5a). Analysis of IL-1β levels in conditioned medium revealed a similar pattern with addition of high extracellular K^+^ completely suppressing IL-1β processing with a significant but not complete inhibition observed upon addition of MKC8866 (Fig. 5b, c).

Following ASC oligomerization pro-caspase-1 is recruited to the inflammasome via CARD:CARD interactions resulting in the generation of active caspase-1 thus enabling pro-IL-1β processing. Any suppression in inflammasome formation would be expected to correlate with a decrease in caspase-1-like activity. Examination of pro-caspase-1 processing via western blotting demonstrated reduced p10 caspase-1 present in the conditioned medium of those THP-1 cells treated with LPS/nigericin plus MKC8866 or 100 mM KCl compared with those treated with LPS/nigericin alone (Fig. 5b). Similarly, addition of MKC8866 efficiently suppressed intracellular caspase-1 like activity as assessed by a flow cytometry-based FLICA assay in LPS/nigericin-treated cells (Fig. 5d). Collectively, these results indicate that selective inhibition of IRE1α RNase activity in THP-1 cells diminishes assembly of the NLRP3 inflammasome and, thereby, reduces processing of pro-IL-1β and release of active p17 IL-1β.

**Fig. 6:**
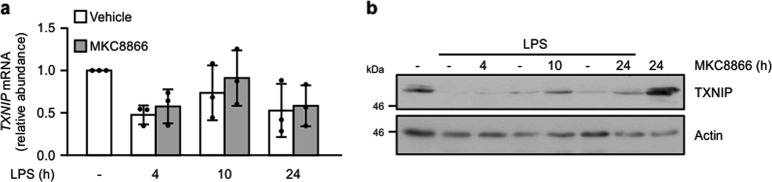
TXNIP expression decreases in THP-1 cells upon LPS treatment. THP-1 cells were treated for the indicated times with 1 μg/ml LPS alone or in combination with 20 μM MKC8866 and after which **a** *TXNIP* expression was determined by Q-PCR (*n* = 3) and **b** by immunoblotting. Error bars represent SD.

Thioredoxin interacting protein (TXNIP) has been demonstrated to bind NLRP3 during oxidative stress leading to increased inflammasome activation^24^. Furthermore, several reports have proposed that IRE1α signaling increases TXNIP expression and, thereby, inflammasome formation^30,31^. Based on these studies, we asked if IRE1α activation promotes inflammasome formation through regulation of TNXIP expression. To answer this, we examined TNXIP expression following inflammasome stimulation in the presence and absence of MKC8866. Rather than observe an increase in TXNIP expression we found that LPS addition led to decreased expression of TXNIP transcript, which was partially suppressed by addition of MKC8866 (Fig. 6a). Likewise, when we examined expression of TXNIP protein, we observed a similar trend with LPS causing decreased TNXIP expression, which again was partially blocked by combination with MKC8866 (Fig. 6b). Therefore, in this experimental setting, it is unlikely that IRE1α RNase signaling promotes inflammasome formation by increasing TXNIP expression.

### MKC8866 reduces inflammasome activation in primary PBMCs

**Fig. 7:**
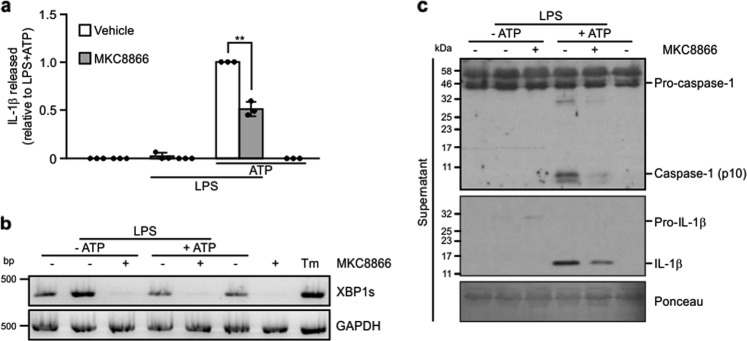
IRE1α RNase inhibition reduces activation of the NLRP3 inflammasome in primary PBMCs. PBMCs isolated from healthy individuals were treated for 2 h with either 0.5 ng/ml LPS alone or in combination with 20 μM MKC8866 followed by addition of 5 mM ATP for 45 min following which cell lysates and conditioned medium was collected. **a** Levels of IL-1β in cell conditioned medium were quantified by ELISA (*n* = 3). **b** IRE1α RNase activity was assessed by monitoring levels of *XBP1* splicing via RT-PCR. Tunicamycin (Tm) treatment served as a positive control for *XBP1* splicing. **c** Immunoblotting for caspase-1 (pro- and p10) and IL-1β (pro- and p17) was performed using PBMC conditioned medium collected following the indicated treatments. *P < 0.05 based on a Student’s *t* test. Error bars represent SD.

To verify that MKC8866 suppression of NLRP3 inflammasome formation is not restricted to THP-1 cells, we isolated PBMCs from healthy adults. Following priming with 0.5 ng/ml LPS for 2 h, primary PBMCs were stimulated with 5 mM ATP for 45 min. Similar to nigericin, ATP triggers K^+^ efflux by selectively activating the P_2_X_7_ receptor^32^. Similar to THP-1 cells, treatment with MKC8866 diminished IL-1β secretion and reduced *XBP1* splicing in primary PBMCs stimulated with LPS and ATP (Fig. 7a, b). Immunoblots using the conditioned medium of LPS/ATP-treated PBMCs confirmed that MKC8866 treatment was associated with reductions in the release of p17 IL-1β and processed pro-caspase-1 (Fig. 7c). Collectively, these results indicate that IRE1α RNase activity also contributes to NLRP3 inflammasome formation in primary human PBMCs.

## Discussion

Classically IRE1α activation is associated with resolution of ER stress via activation of the adaptive, pro-survival UPR. However, an important role for IRE1α signaling is emerging within the innate immune system where it has been linked to the function of several cell types including dendritic cells, macrophages, and natural killer cells^13^. In this study we asked whether IRE1α signaling contributed to activation of the NLRP3 inflammasome in monocytic cells. Similar to the findings reported by Martinon et al., using murine bone marrow derived macrophages (BMDMs)^15^, we observed selective activation of the IRE1α-XBP1 axis in the human monocytic cell line, THP-1 following stimulation with LPS. Addition of signal II, via nigericin treatment, while not altering IRE1α-dependent signaling, significantly enhanced inflammasome activation and pro-IL-1β processing. By selectively inhibiting IRE1α signaling using a small molecule IRE1α RNase inhibitor we observed a substantial reduction in processing of pro-IL-1β in both THP-1 cells and primary PBMCs. Similar to our findings, Tufanli et al. recently reported blocking IRE1α RNase signaling reduced pro-IL-1β processing in PBMCs and NLRP3 inflammasome activation in murine BMDMs^19^ but did not explore the mechanism underpinning these observations.

By assessing the various signaling steps driving NLRP3 inflammasome assembly we demonstrate IRE1α signaling, while not required in THP-1 cells for priming of inflammasome components or induction of pro-IL1β expression, is required for efficient assembly of the NLRP3 inflammasome complex. The reduction we observe in inflammasome assembly, upon IRE1α RNase inhibition, while substantial is not complete. This suggests that IRE1α signaling likely promotes the efficiency of inflammasome assembly by fine-tuning it in some manner but is not an absolute requirement. Similar results were obtained in primary PBMCs where treatment with MKC8866 reduced but did not inhibit pro-IL1β processing and release in response to LPS/ATP treatment.

The signaling pathways initiating structural assembly of the inflammasome are not fully understood, with diverse processes including ion efflux, generation of mitochondrial reactive oxygen species and posttranslational modification of inflammasome components including NLRP3 and ASC implicated^33^. Recent reports have suggested that IRE1α RNase activity can increase NLRP3 inflammasome formation in the pancreatic B cell line INS-1 via regulation of thioredoxin-interacting protein (TXNIP) expression^30,31^. Increased IRE1α RNase activity, induced in INS-1 cells via treatment with pharmacological inducers of ER stress, was demonstrated to degrade miR-17, a microRNA that normally represses TXNIP mRNA resulting in increased TXNIP expression and enhanced inflammasome formation^30^. Similarly, Oslowski et al. reported addition of thapsigargin or tunicamycin to INS-1 cells induced TXNIP expression, which increased levels of pro-IL1β^31^.

In assessing the contribution of TXNIP in our system, we found that LPS treatment decreased TXNIP expression in THP-1 cells. Furthermore, LPS-induced decreases in TXNIP expression were partly blocked upon addition of MKC8866 suggesting that IRE1α-mediated signaling leads to a loss of TXNIP expression in our system. The discrepancy between our work and previously reported results may be a consequence of several factors including the amplitude of IRE1α signaling triggered and the cell types used. We have modeled physiological activation of IRE1α signaling within the innate immune system rather than IRE1α signaling induced by pharmacological inducers of ER stress. Although we have clearly demonstrated a role for IRE1α signaling in fine-tuning assembly of the NLRP3 inflammasome in monocytes following TLR ligation, further studies are required to determine the mechanisms underpinning this observation.

While our results indicate that IRE1α signaling contributes to but is not an essential requirement for inflammasome activation in LPS-stimulated monocytes, it is likely that the importance of this pathway may be heightened in particular settings. Elevated IRE1α signaling, as determined by increased XBP1 splicing, has been reported in monocytes obtained from rheumatoid arthritis and tumor necrosis factor receptor associated periodic syndrome patients^34,35^. Within these specific disease settings, it is plausible that constitutively elevated IRE1α signaling helps drive hyper-stimulation of the inflammasome leading to excessive production of IL-1β. Therefore, selective, targeted use of IRE1α inhibitors could yield benefits in these clinical settings. Indeed, genetic ablation of IRE1α signaling in a mouse model of rheumatoid arthritis has been demonstrated to confer significant therapeutic benefits^18^.

In summary, our study highlights a previously unknown role for IRE1α-dependent signaling in the structural assembly of the NLRP3 inflammasome. We demonstrate blockade of IRE1α signaling, through the use of a small molecule inhibitor, efficiently reduces NLRP3 inflammasome formation and pro-IL1β processing in both THP-1 cells and primary PBMCs. Our findings further support the emergence of IRE1α as a driver of innate immune responses and suggest therapeutically targeting IRE1α could yield clinical benefit in conditions characterized by excessive NLRP3 inflammasome formation.
