# Correction: Inhibition of IRE1α RNase activity reduces NLRP3 inflammasome assembly and processing of pro-IL1β

**DOI:** 10.1038/s41419-019-2189-6

**Published:** 2020-01-06

**Authors:** Aaron Talty, Shane Deegan, Mila Ljujic, Katarzyna Mnich, Serika D. Naicker, Dagmar Quandt, Qingping Zeng, John B. Patterson, Adrienne M. Gorman, Matthew D. Griffin, Afshin Samali, Susan E. Logue

**Affiliations:** 1 Apoptosis Research Centre, National University of Ireland Galway, Galway, Ireland; 2 School of Natural Sciences, National University of Ireland Galway, Galway, Ireland; 3 Regenerative Medicine Institute (REMEDI) at CÚRAM Centre for Research in Medical Devices, School of Medicine, College of Medicine, Nursing and Health Sciences, National University of Ireland Galway, Galway, Ireland; 4 Institute of Anatomy and Cell Biology, Faculty of Medicine, Martin Luther University Halle-Wittenberg, Halle (Saale), Germany; 5 Fosun Orinove PharmaTech Inc., Suite 211, Building A4, 218 Xinghu St., Suzhou Industrial Park, 215123 Jiangsu, China; 6 Fosun Orinove PharmaTech Inc., 3537 Old Conejo Road, Suite 104, Newbury Park, CA 91320 USA; 7 Department of Human Anatomy and Cell Science, Rady Faculty of Health Sciences, Max Rady College of Medicine, University of Manitoba, Winnipeg, MB Canada

**Keywords:** Stress signalling, Inflammasome

**Correction to:Cell Death and Disease**

10.1038/s41419-019-1847-z published online 16 August 2019

In the published version of this paper, an institutional co-affiliation of author Dr. Dagmar Quandt was inadvertently omitted. The following institution should also have been affiliated to the study: Institute of Anatomy and Cell Biology, Faculty of Medicine, Martin Luther University Halle-Wittenberg, Halle (Saale), Germany. This has been corrected in the PDF and HTML versions.

